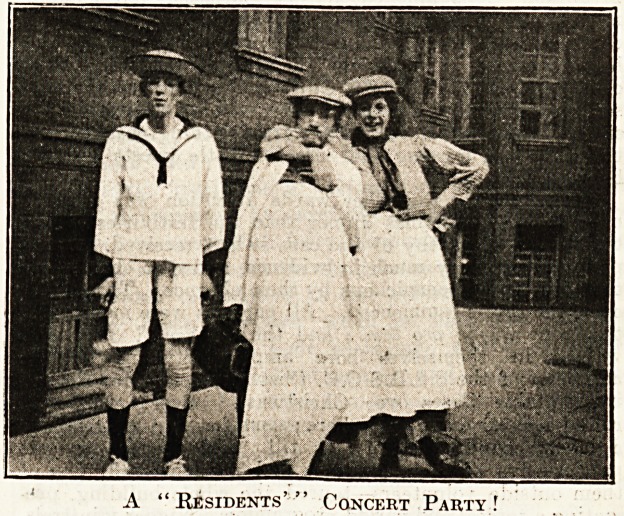# The Hospitals' Christmas

**Published:** 1921-01-01

**Authors:** 


### THE HOSPITALS' CHRISTMAS.

**Last year it was feared that it might not be possible to make much of Christmas in the Hospitals. Not much, that is, if comparison were made with other days gone by, but, nevertheless, 1919 showed most of the old joys coming rapidly back, and this year they have certainly again been with us in full force. On the pages which follow we give such detailed accounts as it has been possible to collect in the short time at our disposal before these pages must go to press. We fear that many stories of Christmas happiness which friends throughout the country are sending must be unavoidably held over until next week. The Voluntary Hospitals' Christmas has been as beautiful, and as representative of a great ideal as the most earnest of their well-wishers could desire. Long may such signs of the true spirit of personal service survive.**

### GUY'S HOSPITAL.

The Christmas season set in with unexampled severity at Guy's. For some weeks dreadful noises springing from the quarters of the resident staff, the house-surgeons and physicians, proclaimed the fact that the famous nigger troupe intended to undergo their 100th, or it may be 1,000th, incarnation.

A Guy's Ward and its Many Balloons.

Charity Ward held the first concert, on Christmas Eve; this was a most successful show. M. Corelli Windeat brought a most excellent band, and many well-known West-End favourites kindly assisted.

On Christmas Day the President of the residents, in a costume anything but academic, began the day by calling on the Superintendent, and wishing him the compliments of the season, after which he led his motley troupe round the wards. As to their voices, some could sing, and the rest could shout, so what more need be said?

After touring the medical wards they went to the out-patients' tea, where some 700 small children were giving, all together, and all at once, a *résumé* of the popular songs of the last twelve months, as a simple aid to digestion.

After this the niggers toured the surgical wards, their enthusiasm increasing as their voices decreased.

As to decorations, these were to suit all tastes. Some wards were just Christmassy, holly, ivy, yes, and mistletoe. Others, avoiding such old-fashioned ideas, were decked with most realistic flowers, convolvuluses, wisteria, poppies. Balloons formed the basis of another scheme, while one had become a country village—each bed had a thatched roof, the bed curtains had latticed windows, and a sign-board near the door gave hopeful but unfounded directions. All the children, and each ward seemed to be full of them, were dressed in special colour scheme to suit the decorations of their ward, and all wore the broadest of grins.

### ST. THOMAS'S HOSPITAL.

Christmas was celebrated at St. Thomas's Hospital in the traditional manner. Every ward was prettily decorated, each ward adopting some special colour scheme carried out in flowers and paper shades on the electric lamps. Presents for the patients were provided privately, and a Christmas dinner of turkey and plum pudding was furnished in all the wards. During the afternoon smoking was permitted in the male wards, and quite enjoyed. Patients' friends were admitted as visitors, and throughout the hospital there was a general air of quiet enjoyment. The treasurer, the Hon. Sir Arthur Stanley, as well as the secretary and the matron, and many of the physicians and surgeons, visited the wards during the day. Fun was provided by house officers and students in fancy costume, culminating in a Christmas-tree in the medical children's ward, where a magnificent Christmas-tree was provided, to which many of the recently discharged children were admitted, where each child was provided with suitable presents after a sumptuous tea, much enjoyed by these little ones. Shortly after four Father Christmas (Dr. H. B. Russell, the Resident Assistant Physician) arrived in his coach, drawn by a magnificent "horse" (two of the resident officers), and proceeded to distribute the presents from the tree. In some of the wards carols were sung by the probationers. During the afternoon an art exhibition, which had been splendidly organised by one of the house surgeons, showed skilled talent of students and nurses. There was an admirable display of water-colours, which is well worth visiting, and where there were so many excellent pictures it is difficult to pick out any for individual mention. In the morning the chapel was crowded to hear the Bishop of Singapore, who had himself recently been a patient in the hospital. The festivities continued during the week. On Monday there was an "At Home" in the Nightingale Home, where a large number of governors, staff, and old Nightingales accepted the invitation of Miss Lloyd Still, the Matron, to the carols. Under the sister of the Preliminary Training School the Nightingale probationers had thoroughly mastered many excellent carols, one or two of the soloists being particularly brilliant in their execution. After the "At Home" a visit was paid to all the wards of the hospital, where a charming rendering was given of

A "Residents'" Concert Party!

**The Hospitals' Christmas** —(*continued*).

different carols, nurses looking very attractive in their uniforms, each bearing a light for reading their music. A party of students was organised, and on Wednesday a first performance of "Whines and Spirits," a delightful medley full of topical songs, was given, details of which we hope to give at further length in our next week's issue. During each afternoon for the remainder of the week the various wards in the hospital were visited, and the week's festivities will conclude on Saturday evening with a second performance in the Governors' Hall.

It is much to be regretted that at the present time four of the wards are unoccupied. Owing to the heavy debt which has accumulated as the result of five years' war work and the amount of restoration which has had to be done, the work of St. Thomas's is seriously cramped for want of funds. A further difficulty is the provision of room for the nursing staff in addition to the great increase in pay which has been given to the nurses, the hours of work having been so reorganised as to bring them within the reasonable limit of fifty-six per week. With the large addition to out-patient work through the development of special departments, it is not possible at the present time without enlargement to provide accommodation for sufficient nurses to staff the wards.

No fixed charge is made to patients at St. Thomas's Hospital, but the great appreciation of the patients of what has been done for them is shown by the fact that in answer to the appeals made by the lady almoners, who put all the circumstances before each individual patient, both in-patient and out-patient, over £250 is being received in contributions from the patients or their friends each week.

### CHRISTMAS AT ST. BARTHOLOMEW'S.

As was fitting on an occasion which "comes but once a year," the celebrations commenced early: and the first item was by no means the least appropriate on the list. Surgery, the emergency ward and Cinderella of the institution, had sent out invitations to eighty children, former patients, for breakfast and a chat with Santa Claus. The Great Hall was decorated on Christmas Eve with evergreens and flags, a Christmas-tree prepared, and tables set for the morning. At 10 A.M. the children trooped in, selected their gifts from the tree, and sat down to a hearty breakfast. This finished, Father Christmas, on a pantomime steed, pranced in to the accompaniment of banging crackers, wails from the musical (?) abominations that they had contained, and shrieks of juvenile laughter. Each child received from him a Christmas gift in the shape of some cunning toy, a kindly word or two, and a shining silver sixpence. Parcels of warm clothing were distributed by the nursing staff ere the little guests left for home.

Meanwhile, in the other wards attention was directed more to the Christmas dinner than the festivities! And the meal was worthy of the care it had received. Plum-pudding was very much in evidence, and diet charts—for that day—were conspicuous by their absence. That safely over, the revels commenced. All patients were moved into the front wards *pro tem.*; and the number of cases who walked in themselves bore ample testimony to the activities of the S.S.R.S.C.C. (Sisters' Society for Retaining Suitable Cases over Christmas). Every ward had raised a piano from somewhere; some few had materialised a Jazz band. From 2 till 5.30 seven concert parties—five of them students and resident staff, two of them outside volunteers—toured the whole building, presenting a topical and varied programme. Nigger minstrels, pierrots, cowboys, and variety turns were all represented, and the performances reached a surprisingly high level of achievement. The bathrooms, as dressing-rooms, had their limitations, in spite of which several good sketches were mounted, including a very topical parody of the "Right to Strike."

The decorations were ubiquitous; but, as was fitting, were nowhere obtrusive. Mistletoe could be seen if you followed the crowds to the dim backwaters, but perhaps the most popular branch was one pendent over the head of a delightful little baby of about eighteen months with truly angelic patience. The shows over, the patients themselves carried on the fun till late at night, and, whilst the revels lasted, their state of mind perhaps was best expressed by the most popular song of the afternoon, "I don't want to get well."

### KING EDWARD VII. HOSPITAL, CARDIFF.

On Christmas Eve the hospital and wards were beautifully decorated, as they always are. A huge Christmas-tree was placed in the centre of Coronation Ward, lit by many coloured electric lamps, and every child in the hospital received an article of clothing and three or four toys, also a large Christmas stocking full of all manner of good things. Father Christmas visited each child, and afterwards went round to the other wards with the Lady Mayoress and a party of ladies, who gave each adult patient a Christmas gift. The matron (Miss Wilson) was responsible for the Christmas-tree, and the presents were supplied by kind friends who always help her.

After the Christmas-tree was over a splendid concert was held in Shand Ward, and all the patients who were able to go from other wards were present at the concert. The hospital and wards are always thrown open to visitors on Christmas Eve, and many took advantage of the permission. The men patients all received pipes, tobacco, and cigarettes, as well as the gifts given by the Lady Mayoress and party.

The patients and babies at the Maternity Hospital in connection with King Edward's also had presents given them. The patients at the Convalescent Home, Lavernock, were provided with gifts.

On Christmas Day all in hospital were provided with roast turkey and full Christmas fare. In most cases the turkeys, &c., were carved by honorary members of the staff. The Lord and Lady Mayoress of Cardiff visited the hospital in the afternoon, and gave flowers in many of the wards. Chocolates, sweets, &c., were provided by kind friends who invariably send us help at Christmas, and a dance for the nursing staff was held on the 28th in the out-patient department, each member of the staff being permitted to invite a friend. Concerts were held in many of the wards during the week.

### ST. MARY'S HOSPITAL.

Carols were sung on Christmas Eve and Christmas morning, and, as usual, the ward decorations were varied and artistic. Tea started at four o'clock, and each patient was allowed to invite one visitor. Excellent entertainments were given by five troupes of medical officers and students, who went from ward to ward carrying their stage properties with them. The opinion of most patients was voiced by the one who said, as he settled down for the night, "This has been a perfect day."

### THE SOUTH LONDON HOSPITAL FOR WOMEN AND CHILDREN.

Carol-singing by the nurses late on Christmas Eve ushered in what proved to be an unusually happy time for the patients at the South London Hospital for Women and Children. Early Christmas morning the resident medical officers, each disguised as Father Christmas, distributed useful and amusing gifts to each inmate, and filled the children's stockings with toys. In the afternoon the gaily-decorated wards, each having its own lighted Christmas-tree, were filled with visitors, each patient having been given leave to invite a friend to tea. On Boxing Day and during the week there were concerts and other entertainments in the wards. The festivities are being brought to a close on New Year's Day by an entertainment provided by members of the nursing staff. The out-patient children's Christmas party, to which a hundred poor children living in the neighbourhood of the Elephant and Castle have been invited, will be held on Saturday, January 8. Gifts of toys for the Christmas-tree, cakes, oranges, and sweets will be most gratefully received by the sister-in-charge, South London Hospital, Out-patient Department, 86-90 Newington Causeway, S.E.

## Figures and Tables

**Figure f1:**
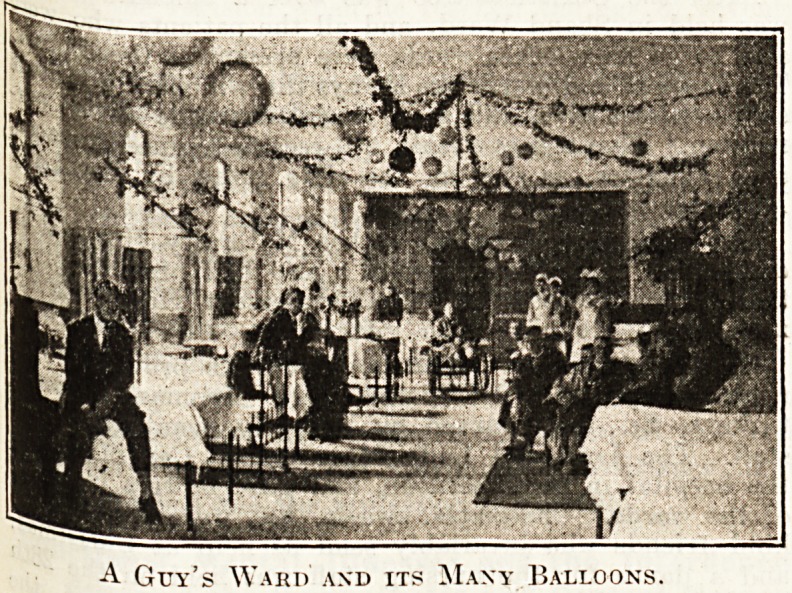


**Figure f2:**